# Are mortality rates similar between jobs in the Queensland coal mine workers’ cohort?

**DOI:** 10.1186/s12889-025-24163-4

**Published:** 2025-09-01

**Authors:** Deborah Catherine Glass, Stella May Gwini, Anthony Del Monaco, Lin Fritschi, Michael J. Abramson, Malcolm Ross Sim, Karen Walker-Bone

**Affiliations:** 1 Faculty of Medicine Nursing and Health Sciences, Monash Centre for Occupational and Environmental Epidemiology, Monash University, 553 St Kilda Road, Melbourne, VIC 3004 Australia; 2 Division of Health Sciences, School of Public Health, Curtin University, Perth, WA Australia

**Keywords:** Coal mine workers, Mortality, Production, Smoking

## Abstract

**Background:**

Coal mine workers are exposed to many occupational hazards which may affect mortality including respirable coal mine dust, crystalline silica and diesel engine emissions. Several studies have shown decreased overall mortality, but studies did not define the jobs held, did not include women coal mine workers and lacked smoking data.

**Methods:**

A cohort of coal mine workers, from Queensland, Australia, was linked to the national death registry. Those who had had a health assessment after 1993 were grouped by job title into eight Work Categories. Mortality by Work Categories were compared to the Australian population to produce standardised mortality ratios (SMRs). Relative mortality ratios (RMR) by sex were calculated comparing risks within the cohort, adjusted for age, era (calendar period) and smoking.

**Results:**

There were 4,555 deaths among 161,534 men and 196 among 23,967 women with job titles.

The median age at inception was 33 (men) and 30 (women) years. SMRs were significantly reduced for men and women. However, compared with other men in the cohort, increased mortality was found from digestive diseases (Maintenance workers and Truck Drivers), respiratory diseases (Construction and Labourers) and accidents (Production, Labourers and Truck Drivers). Circulatory disease mortality was increased for male Production workers adjusted RMR (aRMR) 1.22(95%CI 1.06–1.40), Unexposed Non-Office aRMR 1.64(95%CI 1.15–2.35) and Labourers aRMR 1.48 95%CI (1.00-2.17) and Truck Drivers aRMR 1.21(95%CI 0.95–1.54), while accidental deaths were higher in female cleaners aRMR 3.13(95%CI 1.30–7.57). The suicide rate was higher for men and women in Production jobs.

**Conclusions:**

Although a relatively young cohort, mortality risk varied by type of work. Risk of death from circulatory causes was increased in some workers, even after adjusting for smoking. Suicide rates are higher for men and women doing Production jobs.

**Supplementary Information:**

The online version contains supplementary material available at 10.1186/s12889-025-24163-4.

## Background

Coal mine workers are exposed to many occupational hazards which may affect mortality including respirable coal mine dust, crystalline silica and diesel engine emissions [1, 2]. A recent meta-analysis of 36 studies of male coal mine workers [3] showed decreased overall mortality. This is likely a result of the well known healthy worker effect [4]. Unsurprisingly, there was a 26% increased risk of mortality from non-malignant respiratory disease such as coal miners’ pneumoconiosis in coal mine workers compared with non-coal miners [3]. The review identified several limitations of the literature, including that many studies had short follow-up; failed to describe the type of mine; did not define actual jobs performed; did not include women coal miners; lacked smoking data; and even the most recent cohort was only followed up until 2006.

Following a 1982 survey of the prevalence of coal workers’ pneumoconiosis in Queensland Australia [5], all coal mine workers starting a job in the Queensland coal mining industry were mandated to have pre-employment medicals. The Queensland legislation was expanded in 1993 to require that all coal mine workers receive 5-yearly repeat medical assessments. Information from the assessments was centrally collated, a role currently undertaken by Resources, Safety and Health Queensland (RSHQ), a government agency. The data are available for research and health surveillance purposes and were used to form a cohort.

Coal mine workers were employed at 106 different mine sites over the cohort follow-up period, the majority were open cut. The coal mined in this study was almost all bituminous. Only 5 of the mines had some semi anthracitic deposits. In terms of products, 55 sites supplied coking coal, 49 thermal coal and 15 pulverised coal for injection (PCI). More than one type of product was supplied by 34 sites.

This paper explores whether any major causes of mortality were higher than expected among Queensland coal mine workers in different Work Categories compared to the national population, and to identify whether there were any differences in mortality between workers doing different jobs.

## Methods

We conducted a retrospective cohort study to understand whether mortality varied by type of mine work. The cohort formation and overall mortality has been described previously [6]. Briefly, personal identifiers, smoking status, date of assessment, job title (for assessments after 1993) and mine name were provided by RSHQ from the data collected at each medical assessment.

At each assessment, workers were asked whether or not they were smokers and the smoking status as recorded at their most recent assessment was used for this study. Workers who were recorded as not smoking at all assessments were classified as never smokers; those who were smoking at the most recent assessment were classified as current smokers whilst those who said they were not smoking at the recent assessment but had been recorded as smoking at a previous assessment were deemed ex-smokers. No health data were included in the information received from RSHQ.

Coal mine workers were employed at 106 different mine sites over the cohort follow-up period, the majority were open cut. The coal mines in this study were almost all bituminous. Only 5 of the mines had some semi anthracitic deposits. In terms of products, 55 sites supplied coking coal, 49 thermal coal and 15 pulverised coal for injection (PCI). 34 sites supplied more than one type of product.

**Fig. 1:**
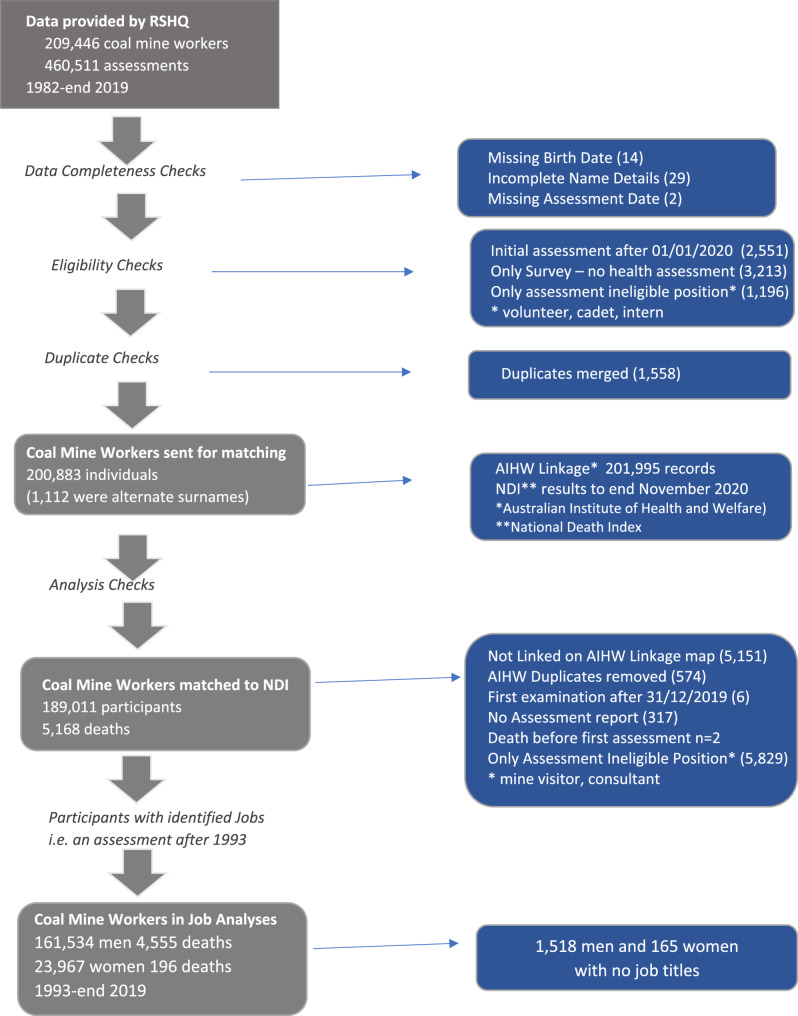
Cohort Structure showing number of coal mine workers and flow of data and exclusions (Individuals may have been excluded for more than one reason)

The cohort was assembled using assessment data collected after the 1982 survey, however the data presented in this paper, relate only to members of the cohort with a health assessment from 1993 because no job titles were recorded prior to this date. There were 1,518 men and 165 women with no job titles recorded at any assessments since 1993 and these were excluded from this analysis. Cohort person years were counted from the first assessment on or after 1 January 1993 and they ceased to contribute person-years at the date of death or 30/11/2020 whichever was earlier. Figure 1 presents the structure of the cohort.

A large number of unique job titles provided by the workers (49,490) were identified and coded to one of 42 job groups using inclusion and exclusion search terms by an experienced occupational hygienist (DCG) and data manager (ADM). These job groups reflected various roles in mining and were then amalgamated into eight Work Categories that were agreed upon in consultation with the data custodians, industry and worker representatives. These Work Categories were: Production (including miners, operators), Maintenance (electricians, fitters, etc.), Administration, Unexposed Non-Office (e.g. control room, environmental services), Occasionally Exposed (e.g. engineers, surveyors), Exploration Drillers, Construction or “Unclear”. Where possible, Labourers, Cleaners, Truck Drivers and Supervisors were allocated to a Work Category such as Production or Maintenance. However, some of these workers could not be allocated to any of the Work Categories and thus formed the “Unclear” Work Category. For example, it was impossible in some cases to ascertain whether a cleaner was cleaning offices or was an industrial cleaner (see supplementary file) Workers were excluded from the cohort if the only job-related information showed that they were a mine site visitor, consultant, vacation student, cadet or intern, because they were not regarded as doing regular coal mine work (*n* = 5,829).

Work Categories were analysed as ‘Ever’ except for Administration, Unexposed Non-Office and Occasionally Exposed categories where workers were categorized into these categories only if all of their recorded job titles were within that category.

Deceased workers were identified by the Australian Institute of Health and Welfare (AIHW) linkage of the cohort to the Australian National Death Index (NDI) [7] via the National Linkage Map. However, 5,151 (3%) individuals were excluded because they could not be linked through the map, which is based on registration for Australian universal health care. At the time of linkage, the NDI was complete from 01/01/1980 until 30/11/2020 for cause of death data, coded to ICD 10. Matching to the Australian national data was necessary because some workers were not resident in Queensland. Coal mine workers may be Fly-in Fly-out or retire to other states so that matching only to the Queensland state data would have underestimated the mortality and resulted in loss to follow-up.

The cohort mortality rates were compared to the Australian national population rates deriving Standardised Mortality Ratios (SMR) which were standardised for age and era. Australian population mortality data were obtained from the General Record of Incidence and Mortality (GRIM) books [8]. The mortality of workers in each Work Category was also compared to the rest of the cohort deriving relative mortality ratios (RMR). Separate risks were calculated for men and women. Additionally, external causes of death (including suicides and accidents) were compared with state of Queensland rates (provided by the Australian Bureau of Statistics) because rates of suicide and accidental death in Queensland are higher than in the rest of Australia [9, 10]. 

The mortality of men and women were examined separately because they may respond differently to exposures. The combined analyses would likely only reflect effects in men because there are six times more men in the study than women [11]. 

SMRs were estimated using the Stata command *strate*. Smoking data were used, together with age and era in Poisson regression models to adjust the calculated RMRs. Two-way interactions between age and smoking status were explored in the base model and only statistically significant interactions included in subsequent models. Data were analysed using Stata Statistical Package version 18 (StataCorp. 2023. Stata Statistical Software: Release 18. College Station, TX: StataCorp LLC). To preserve privacy, cells with fewer than 6 deaths have been reported as < 6.

The study was granted ethics approval including a waiver of individual consent by the Human Research Ethics Committees of Monash University (Study number 22729) and the AIHW, in accordance with the Declaration of Helsinki.

## Results

The cohort included 161,534 men and 23,967 women had experienced a health assessment from 1993 onwards and had a job title recorded. There were 4,555 deaths among men and 196 deaths among women over 1,953,032 and 238,437 person-years, respectively.

Table 1 summarizes the distribution of workers in this analysis, by Work Categories. As shown, 48% of men and 61% of women had their first assessment in or after 2010, but the proportions vary somewhat by Work Category. About half of these workers only had one assessment (*n* = 98,023; 52.8%) and 29.2% (*n* = 37,094) had two or three assessments.

**Table 1:** Description of the men and women in the cohort who had assessments after 1993 by Work Category

Work Category*	*N* (%)	Median age at 1 st assessment	Ever smoked *n* (%)**	Period of first assessment after 1993 (row %)			Number of deaths (%)
				1993–1999	2000–2009	2010 +	
ALL MEN *N* = 161,534	---	32.6	86,787 (54.1)	9.6	42.5	47.9	4,555 (100.0)
ONLY administration	12,596 (7.7)	40.6	5,319 (42.5)	4.5	35.1	60.3	295 (2.3)
ONLY unexposed Non-Office	4,494 (2.8)	34.2	2,375 (53.3)	2.9	24.3	72.8	122 (2.7)
ONLY occasionally exposed	10,714 (6.6)	29.8	3,381 (31.8)	3.0	36.21	60.8	144 (1.3)
EVER maintenance- all	61,309 (37.6)	29.9	32,731 (53.7)	9.7	45.7	44.6	1,391 (2.3)
EVER production- all	55,252 (33.9)	32.9	33,627 (61.2)	16.7	45.1	38.1	1,979 (3.6)
EVER exploration	4,970 (3.1)	29.2	3,064 (62.0)	0.9	44.1	55.0	80 (1.6)
EVER construction	9,034 (5.5)	32.5	5,416 (60.3)	3.1	41.8	55.1	254 (2.8)
Unclear work category							
EVER labourer	5,814 (3.6)	27.6	3,707 (64.0)	2.3	55.2	42.5	138 (2.4)
EVER cleaner	948 (0.6)	29.2	606 (64.5)	3.2	42.5	54.3	21 (2.2)
EVER supervisor	5,575 (3.4)	36.1	3,517 (63.4)	19.7	53.5	26.8	163 (2.9)
EVER truck driver	7,701 (4.7)	40.5	4,881 (63.8)	7.2	53.0	39.8	336 (4.4)
ALL WOMEN *N* = 23,967	---	30.1	10,934 (45.6)	3.2	35.6	61.2	196 (100.0)
ONLY administration	7,538 (31.2)	30.1	2,801 (37.5)	5.2	34.9	59.9	50 (0.7)
ONLY unexposed non-office	2,049 (8.5)	32.4	1,019 (50.1)	1.6	24.8	73.6	20 (1.0)
ONLY occasionally exposed	1,874 (7.8)	26.6	656 (35.3)	1.7	23.5	74.8	9 (0.5)
EVER maintenance- all	2,025 (8.4)	28.7	1,042 (51.9)	3.0	29.8	67.2	9 (0.4)
EVER production- all	6,913 (26.6)	30.2	3,543 (51.6)	1.8	38.5	59.7	51 (0.7)
EVER exploration	43 (0.2)	29.9	20 (46.5)	0.0	39.5	60.5	0
EVER construction	113 (0.5)	29.3	61 (54.0)	2.7	38.9	58.4	< 6
Unclear work category							
EVER labourer	245 (1.0)	31.9	145 (59.9)	N/A	59.6	40.4	< 6
EVER cleaner	2,763 (11.4)	36.2	1,686 (61.5)	0.5	48.3	51.2	48 (1.7)
EVER supervisor	66 (0.3)	37.0	36 (54.5)	1.5	53.0	45.5	0
EVER truck driver	894 (3.7)	35.2	512 (57.6)	1.2	54.9	43.9	6 (0.7)

*Individuals may appear in more than one Work Category if they have held (for example) both Maintenance and Production jobs so the number of deaths column will not total to total number of deaths in the cohort

**Smoking status not recorded for 191 women and 1,048 men. Percentages are calculated from workers whose smoking status was known

Not all members of the cohort had recorded more than one assessment at least five years after their initial assessment. Only 11% of Administration workers had more than one assessment. About 40% of male workers in Maintenance jobs, 60% of male Production workers had more than one assessment. The Production workers more than one assessment included 81% of CHPP workers (Coal Handling and Preparation Plant workers), 86% of ERZ/Deputies (Explosion Risk Zone controller) and 96% of OCE (Open Cut Examiner) and Dragline operators. The proportions with more than one assessment were lower for female workers.

Over a third of men (38%) had held at least one job in Maintenance, 34% had a job in the Production, while 31% of women had only worked in Administration and 27% of women had held one or more jobs in the Production. About two-thirds of workers across all Work Categories had other job titles within the same category, the highest proportions were reported in the Maintenance (71.4%) and Construction (70.3%) Work Categories (data presented in the supplementary file). Half of the workers who were ever Labourers (53.6%) or ever Truck Drivers (50.2%) had only been in that job group, whilst 27% of Supervisors only had that job group recorded.

Median age at first examination (after 1993) ranged from 27 years (Labourers in the Unclear Work Category) to 41 years (Administration workers and for Truck Drivers in the Unclear Work Category) among men and 26 (Occasionally exposed workers) to 37 (Supervisors) years in women (Table 1).

Smoking status was recorded for more than 99% of participants. Approximately half the cohort had ever smoked with differing proportions between Work Categories (Table 1). About half of the ever smokers reported quitting i.e. were ex-smokers.

### All-cause mortality comparisons for men and women

**Fig. 2:**
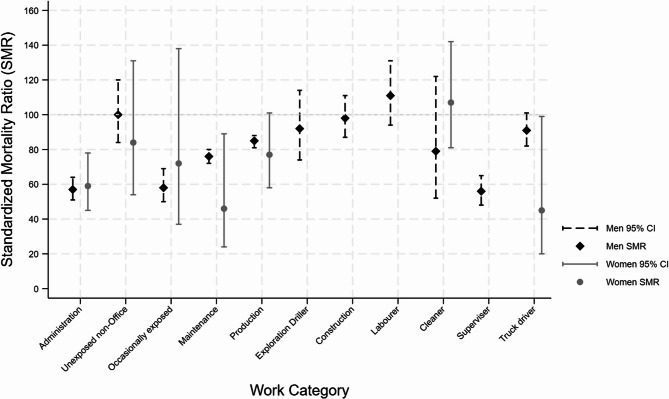
Summary of standardised mortality ratios for all causes of death combined, by sex and Work Category; comparison with the general Australian population rates. The Labourer, Cleaner, Supervisor and Truck Driver groups refer those in the Unclear Work Category [Note that no estimates are provided for women in the Exploration Driller, Construction, Labourer, Supervisor or Truck driver groups because of small numbers]

Figure 2 illustrates the all-cause mortality SMRs compared to the general Australian population, by Work Categories. There were few women in some Work Categories, so mortality rates were compared to population rates only for selected Work Categories. The confidence intervals for All Cause SMRs overlapped for men and women in most Work Categories, although the confidence intervals were wider for women.

Comparisons within the cohort after adjusting for age, era and smoking status, showed increased all-cause aRMRs for men in the Unexposed Non-office, Production, and Construction Work Categories and for male Labourers, male Truck Drivers and female Cleaners in the Unclear Work Category (Table 2). Of these, only the findings for male Labourers and Truck Drivers were statistically significant. Male workers in the following Work Categories had significantly reduced overall relative mortality: only Administration, only Occasionally Exposed jobs, ever Maintenance, and Supervisors (Table 2).

**Table 2:** Comparison of all-cause mortality rates between workers in each of the work categories vs. all other workers not in the work category, separately for men and women

Work Category	MEN		WOMEN	
	Reference: all other male workers		Reference: all other female workers	
	Unadjusted RMR (95%CI)	Adjusted* RMR (95%CI)	Unadjusted RMR (95%CI)	Adjusted* RMR (95%CI)
ONLY administration	0.97 (0.86–1.09)	0.76 (0.68–0.86)	0.67 (0.49–0.92)	0.81 (0.59–1.12)
ONLY unexposed non-office	1.31 (1.09–1.56)	1.15 (0.95–1.38)	1.34 (0.84–2.12)	1.08 (0.67–1.73)
ONLY occasionally exposed	0.56 (0.47–0.66)	0.80 (0.68–0.95)	0.65 (0.33–1.27)	1.06 (0.54–2.07)
EVER maintenance	0.69 (0.65–0.73)	0.91 (0.86–0.97)	0.58 (0.30–1.13)	0.58 (0.30–1.13)
EVER production	1.17 (1.10–1.24)	1.04 (0.97–1.10)	0.90 (0.65–1.23)	1.04 (0.75–1.43)
EVER exploration	0.64 (0.52–0.80)	0.98 (0.78–1.22)	na	na
EVER construction	1.12 (0.99–1.27)	1.10 (0.96–1.25)	< 6	< 6
Unclear work category				
EVER labourer	0.87 (0.74–1.04)	1.24 (1.05–1.47)	< 6	< 6
EVER cleaner	0.90 (0.59–1.37)	0.84 (0.54–1.29)	2.21 (1.60–3.05)	1.34 (0.97–1.87)
EVER supervisor	0.78 (0.67–0.91)	0.68 (0.58–0.80)	na	na
EVER truck driver	1.53 (1.37–1.71)	1.12 (1.00–1.25)	< 6	< 6

*Adjusted for era, age, smoking status, age * era interaction, age * smoking status interaction

na = not applicable either no worker in category or no deaths recorded in category

### Major causes of death for female coal mine workers

Increased risks of accidental death and suicide were seen for women in some Work Categories: excess suicides in Production workers (SMR 201, 95%CI 108–374; aRMR 3.48, 95%CI 1.25–9.67 *n* = 10) and deaths from accidents among Cleaners (SMR 276, 95%CI 138–552); RMR 3.13, 95%CI 1.30–7.57, *n* = 8) (Tables 3 and 4). For most categories of death, the rates were similar to the Australian and Queensland rates. However, the SMR compared to Queensland (SMR_Q_) for suicides among women cleaners was somewhat attenuated (SMR_Q_ 177, 95%CI 95–329). Deaths in this group from accidents were still increased (SMR_Q_ 263, 95%CI 132–526) (Table 3). Deaths from malignancies were not increased for women in any Work Category.

**Table 3:** Standardised mortality ratios (SMR) for female coal mine workers by work category compared to the Australian and Queensland populations

Cause of Death Categories**	Observed	Comparison rate			
		Australia		Queensland	
		E	SMR (95% CI)	E	SMR_Q_ (95% CI)
Only administration *N=7,538; PY=79,488*					
All causes	50	85	59 (45–78)	88	57 (43–75)
All malignancies	30	38	79 (55–113)	38	78 (55–112)
All injury and trauma	< 6		14 (3–54)		13 (3–51)
Accidents	< 6		14 (2–97)		13 (2–92)
Suicide	0				
Only unexposed non-Office* N=2,049; PY=18,448*					
All causes	20	24	84 (54–131)	24	82 (53–127)
All malignancies	8	11	72 (36–144)	11	71 (36–142)
All injury and trauma	< 6		145 (60–348)		137 (57–329)
Accidents	< 6		116 (29–464)		111 (28–445)
Suicide	< 6		144 (36–575)		128 (32–511)
Ever maintenance* N=2,025; PY= 17,909*					
All causes	9	19	46 (24–89)	20	45 (23–86)
All malignancies	< 6		47 (18–126)		47 (17–124)
All injury and trauma	< 6		61 (15–242)		57 (14–226)
Accidents	< 6		121 (30–486)		117 (29–466)
Suicide	0				
Ever Production* N=6,913; PY=66,047*					
All causes	51	66	77 (58–101)	69	74 (56–98)
All malignancies	22	29	75 (49–114)	30	74 (49–112)
All injury and trauma	16	12	131 (80–214)	13	122 (75–200)
Accidents	6	6	100 (45–222)	6	95 (43–212)
Suicide	10	5	201 (108–374)	6	177 (95–329)
Ever cleaner in unclear work category *N=2,763; PY=30,034*					
All causes	48	45	107 (81–142)	46	105 (79–139)
All malignancies	21	22	97 (63–149)	22	96 (63–148)
All injury and trauma	15	6	261 (157–433)	6	247 (149–409)
Accidents	8	3	276 (138–552)	3	263 (132–526)
Suicide	< 6		177 (66–471)	3	156 (59–417)

Abbreviations: PY is Person Years, E is expected, SMR_Q_ is Standardized Mortality Ratios with Queensland population rates as the reference

**ICD Codes: All Causes A00 - Z99; All Malignancies C00–C97, D45–D46, D47.1, D47.3, D47.4, D47.5

All Injury and Trauma V01-Y98; Accidents V01-X59, Y85-Y86; Suicide X60-X84

**Table 4:** Relative mortality ratio (RMRs†) for female coal mine workers by work category compared to all other female workers

Cause of Death**	Unadjusted RMR (95% CI)	Adjusted* RMR (95% CI)
Only administration		
All causes of death	0.67 (0.49–0.92)	0.81 (0.59–1.12)
All malignancies	0.94 (0.61–1.45)	1.15 (0.74–1.79)
All circulatory	0.97 (0.44–2.14)	1.21 (0.55–2.67)
All injury & trauma	0.10 (0.02–0.41)	0.12 (0.03–0.49)
Accidents	0.10 (0.01–0.76)	0.12 (0.02–0.96)
Suicide	-	-
Ever production		
All causes of death	0.90 (0.65–1.23)	1.04 (0.75–1.43)
All malignancies	0.80 (0.50–1.29)	1.00 (0.62–1.61)
All circulatory	0.89 (0.38–2.09)	1.07 (0.43–2.64)
All injury & trauma	1.58 (0.85–2.93)	1.51 (0.79–2.87)
Accidents	1.06 (0.41–2.74)	1.06 (0.39–2.87)
Suicide	3.80 (1.45–9.98)	3.48 (1.25–9.67)
Ever cleaner in unclear work category		
All causes of death	2.21 (1.60–3.05)	1.34 (0.97–1.87)
All malignancies	2.01 (1.24–3.26)	1.14 (0.70–1.85)
All circulatory	0.85 (0.26–2.81)	0.48 (0.15–1.57)
All injury & trauma	3.80 (2.03–7.11)	2.89 (1.53–5.45)
Accidents	4.36 (1.81–10.52)	3.13 (1.30–7.57)
Suicide	2.18 (0.71–6.69)	1.80 (0.56–5.74)

†Risk in each work category was compared to the rest of the female cohort

*Adjusted for era of first examination, age, smoking status, age & era interaction and age & smoking status interaction

**ICD Codes: All Causes A00 - Z99; All Malignancies C00–C97, D45–D46, D47.1, D47.3, D47.4, D47.5; All circulatory I00 – I99; All Injury and Trauma V01-Y98; Accidents V01-X59, Y85-Y86; Suicide X60-X84

### Major causes of death for male coal mine workers

**Fig. 3:**
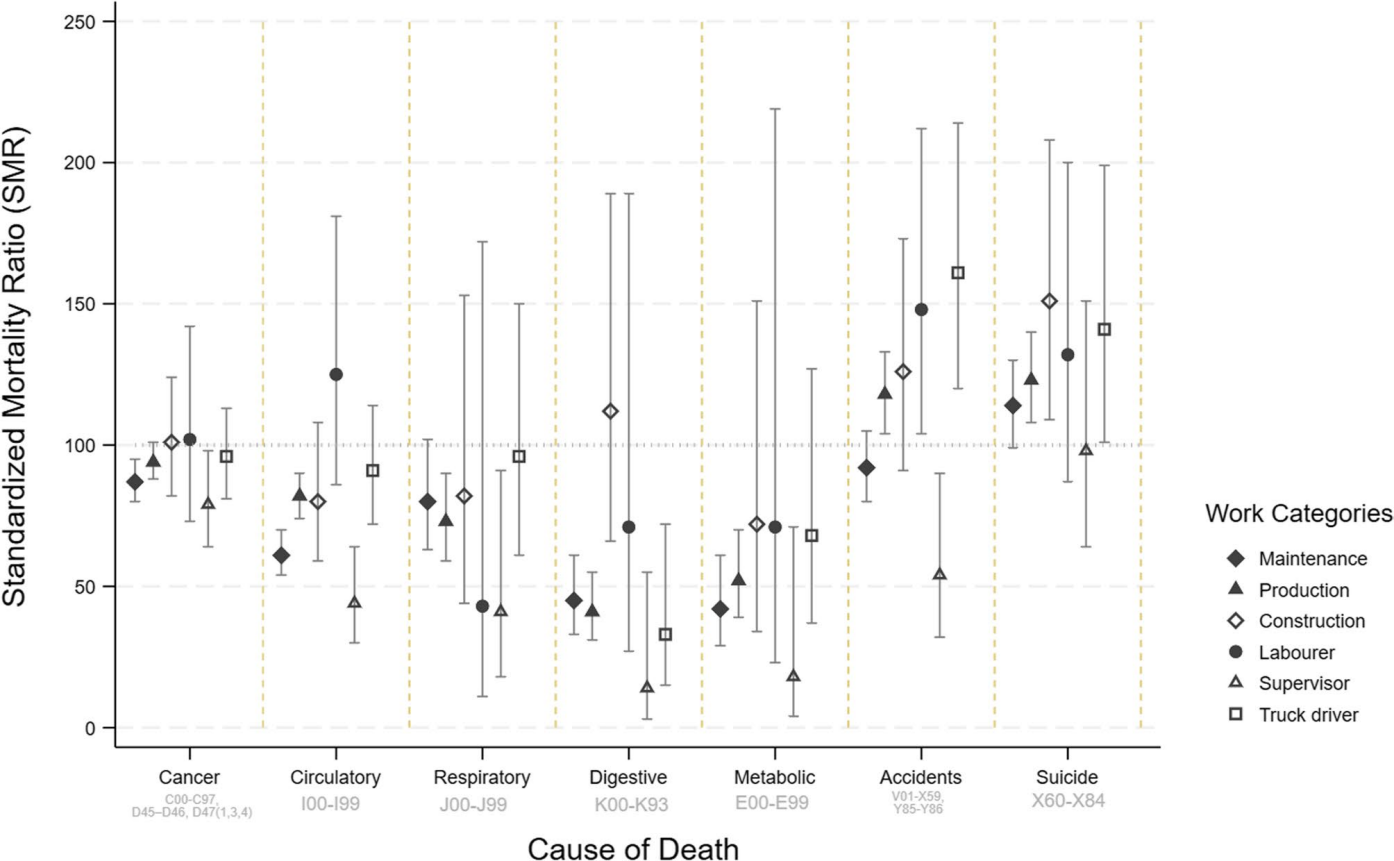
Summary of standardised mortality ratios for selected causes of death (ICD10 codes included) among male coal mine workers by whether they were ever in the selected Work Categories. The Labourer, Supervisor and Truck Driver groups refer those in the Unclear Work Category. [Exploration Drillers and Cleaners omitted because of the small number of deaths], comparison with the general Australian population rates

**Fig. 4:**
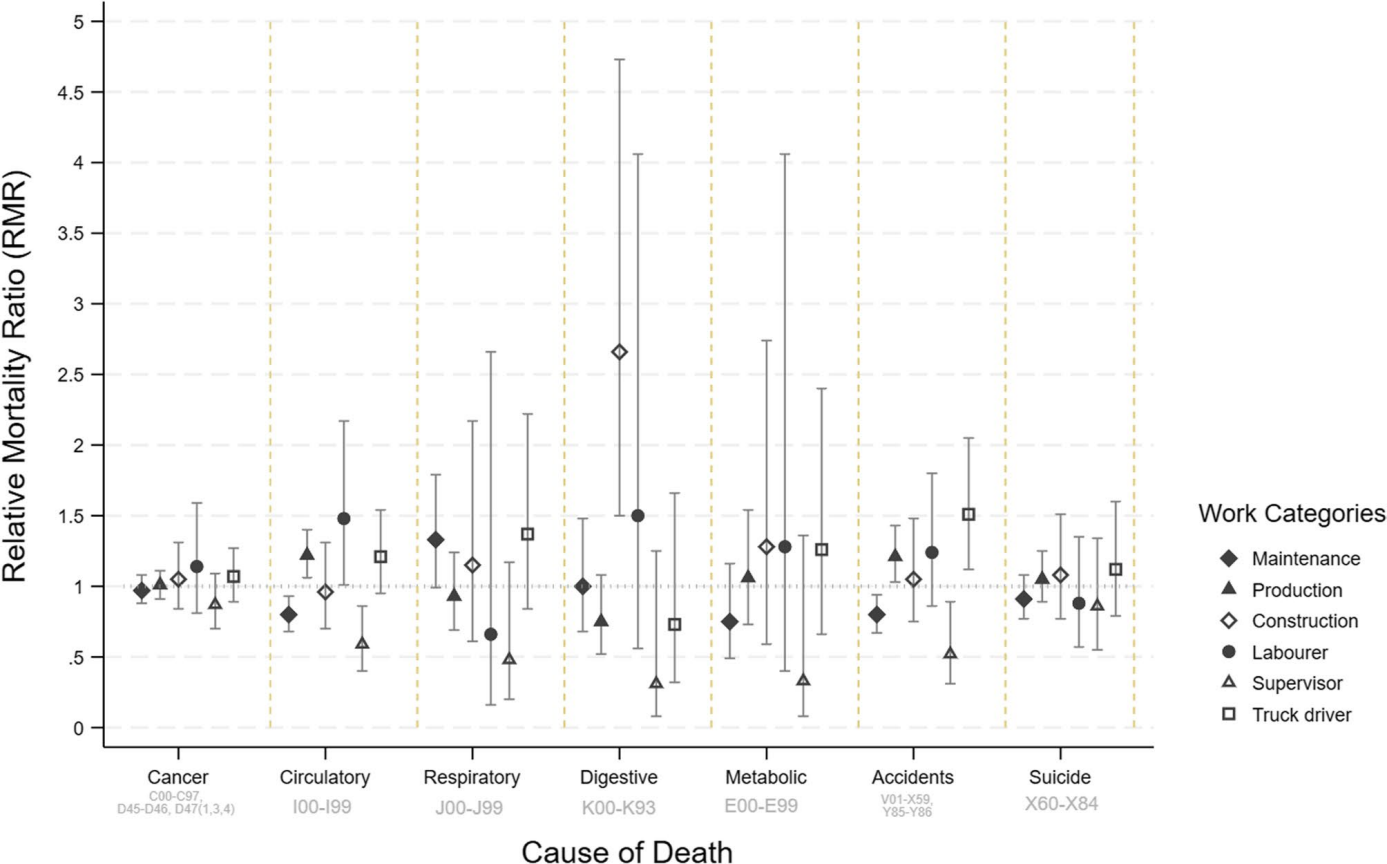
Summary of relative mortality ratios (adjusted for era, age and smoking status) for selected causes of death (ICD-10 codes included) among male coal mine workers by whether they were ever in the selected Work Categories. The Labourer, Supervisor and Truck Driver groups refer those in the Unclear Work Category. [Exploration Drillers and Cleaners omitted because of the small number of deaths] compared to all other categories

Mortality from specific major causes of death (except from accidents and suicides) within Work Categories among men were either lower than, or similar to, the age-standardised population rates (Fig. 3; Table 5). Comparisons within the cohort, RMRs adjusted for age, era and smoking status (aRMR), showed differences in risk by Work Category (Fig. 4). Figures 3 and 4 show that Supervisors had the lowest SMRs and RMRs for all major causes of death. The SMRs for all cancers combined showed some variability between Work Categories (Fig. 3) although the confidence intervals overlapped, however the inter-category variability was reduced for RMRs after adjusting for smoking (Fig. 4).

**Table 5:** Standardised mortality ratios (SMR) for male coal mine workers by work category compared to the Australian and Queensland populations

Cause of Death Ccategories**	Observed	Comparison rate			
		Australia		Queensland	
		E	SMR (95% CI)	E	SMR_Q_ (95% CI)
Only Administration *N=12,596; PY=132,633*					
All causes	295	515	57 (51–64)	530	56 (50–62)
All malignancies	140	192	73 (62–86)	201	70 (59–82)
All injury and trauma	44	79	56 (42–75)	87	50 (38–68)
Accidents	17	40	43 (27–69)	43	40 (25–64)
Suicide	25	33	77 (52–113)	40	62 (42–92)
Only unexposed non-Office *N=4,494; PY=41,033*					
All causes	122	122	100 (84–120)	127	96 (81–115)
All malignancies	45	42	107 (80–143)	44	102 (76–137)
All injury and trauma	22	24	92 (61–140)	27	81 (54–124)
Accidents	12	12	100 (57–176)	13	92 (52–162)
Suicide	10	10	100 (54–185)	13	79 (43–148)
Only occasionally exposed *N=10,714; PY= 111,574*					
All causes	144	247	58 (50–69)	259	56 (47–65)
All malignancies	62	77	81 (63–103)	80	77 (60–99)
All injury and trauma	41	65	63 (47–86)	74	55 (41–75)
Accidents	16	33	49 (30–80)	36	45 (28–73)
Suicide	24	27	88 (59–131)	34	70 (47–104)
Ever maintenance *N=61,309; PY= 758,949*					
All causes	1391	1836	76 (72–80)	1915	73 (69–77)
All malignancies	514	592	87 (80–95)	618	83 (76–91)
All injury and trauma	434	445	98 (89–107)	502	86 (79–95)
Accidents	207	225	92 (80–105)	245	84 (74–97)
Suicide	211	185	114 (99–130)	230	92 (80–105)
Ever production *N=55,252; PY=753,893*					
All causes	1979	2339	85 (81–88)	2422	82 (78–85)
All malignancies	771	821	94 (88–101)	858	90 (84–96)
All injury and trauma	509	447	114 (104–124)	500	102 (93–111)
Accidents	267	227	118 (104–133)	246	109 (96–122)
Suicide	228	185	123 (108–140)	227	101 (88–115)
Ever construction *N= 9,034; PY= 100,766*					
All causes	254	259	98 (87–111)	270	94 (83–106)
All malignancies	87	86	101 (82–124)	90	97 (78–119)
All injury and trauma	77	59	132 (105–164)	66	116 (93–145)
Accidents	37	29	126 (91–173)	32	116 (84–159)
Suicide	37	25	151 (109–208)	31	121 (87–169)
Ever exploration Driller *N= 4,970; PY= 54,690*					
All causes	80	87	92 (74–114)	93	86 (69–107)
All malignancies	19	22	88 (56–137)	23	84 (54–132)
All injury and trauma	43	32	136 (101–184)	36	119 (88–160)
Accidents	23	16	146 (97–219)	17	133 (88–199)
Suicide	15	13	112 (67–185)	17	88 (53–146)
Unclear work category					
Ever labourer *N=5,814; PY=69,441*					
All causes	138	124	111 (94–131)	131	105 (89–124)
All malignancies	35	34	102 (73–142)	36	98 (70–136)
All injury and trauma	56	40	140 (108–182)	46	123 (94–159)
Accidents	30	20	148 (104–212)	22	135 (95–193)
Suicide	22	17	132 (87–200)	21	104 (69–159)
Ever cleaner *N=948; PY=10,112*					
All causes	21	26	79 (52–122)	28	76 (50–117)
All malignancies	6	9	67 (30–149)	9	64 (29–142)
All injury and trauma	9	6	158 (82–303)	7	139 (72–266)
Accidents	7	3	242 (115–507)	3	221 (106–464)
Suicide	< 6		84 (21–334)		67 (17–266)
Ever supervisor *N=5,575; PY=85,840*					
All causes	163	293	56 (48–65)	302	54 (46–63)
All malignancies	83	105	79 (64–98)	110	75 (61–94)
All injury and trauma	35	52	68 (49–94)	57	61 (44–85)
Accidents	14	26	54 (32–90)	28	50 (29–84)
Suicide	21	21	98 (64–151)	26	81 (53–125)
Ever truck driver* N=7,701; PY=96,203*					
All causes	336	369	91 (82–101)	380	88 (79–98)
All malignancies	133	139	96 (81–113)	145	92 (77–109)
All injury and trauma	80	57	141 (113–176)	63	128 (103–159)
Accidents	46	29	161 (120–214)	31	149 (112–199)
Suicide	33	23	141 (101–199)	28	116 (83–164)

Abbreviations: PY is Person Years, E is expected, SMR_Q_ is Standardized Mortality Ratios with Queensland population rates as the reference

**ICD Codes: All Causes A00 - Z99; All Malignancies C00–C97, D45–D46, D47.1, D47.3, D47.4, D47.5

All Injury and Trauma V01-Y98; Accidents V01-X59, Y85-Y86; Suicide X60-X84

Higher lung cancer mortality was observed among male Production (SMR 115, 95%CI 100–132; aRMR 1.14, 95%CI 0.93–1.40, *n* = 194) and Construction workers (SMR 173, 95%CI 121–248; aRMR 1.60, 95%CI 1.09–2.34, *n* = 30) but not in other Work Categories including Maintenance (SMR 106, 95%CI 75–109; aRMR 0.87, 95%CI 0.69–1.09). Within Production, lung cancer mortality was higher for those who ever worked as Drillers (general) (SMR 210, 95%CI 128–342, *n* = 16) or Operators (SMR 118, 95% 101–139, *n* = 153) compared to the general population. Lung cancer mortality showed a monotonic increase with time since first assessment among Production workers. Male coal mine workers aged under 65 and those over 65 had similar patterns of overall mortality, however lung cancer deaths were higher than expected for older men (65+) but not for younger men (< 65 years) in Production and Construction Work Categories (Table 6).

**Table 6:** Lung cancer (ICD codes C33-C34) deaths, number and SMR by age and work category for men

	Work Category					
	Only Administration	Only Unexposed Non-Office	Only Occasionally Exposed	Ever Production	Ever Maintenance	Ever Construction
Total number of lung cancer deaths	30	< 6	14	194	106	30
Comparisons with Australian population by AGE [SMR (95%CI)]						
<65	46 (26–84)	40 (10–158)	82 (41–164)	103 (85–124)	79 (62–101)	156 (99–248)
65+	116 (74–183)	84 (27–260)	114 (51–253)	134 (108–166)	114 (84–153)	207 (117–364)
Australian population comparisons by TIME SINCE 1st ASSESSMENT [SMR (95%CI)]						
≤5 years	51 (21–123)	43 (6–307)	27 (4–195)	74 (46–119)	80 (48–132)	136 (57–327)
>5–10 years	66 (34–126)	33 (5–235)	97 (40–233)	104 (76–142)	79 (53–119)	191 (106–345)
> 10–15 years	85 (42–169)	175 (56–542)	134 (56–322)	125 (95–165)	98 (69–140)	214 (115–398)
>15 years	112 (56–224)	No deaths	119 (38–367)	128 (104–157)	98 (71–135)	125 (47–333)

The non-malignant respiratory disease mortality rate, across all Work Categories, was either comparable to or lower than the expected rates when compared to the national population (Fig. 3). On the other hand, within cohort comparisons showed that the risk of non-malignant respiratory disease was raised (but did not reach statistical significance) for Maintenance workers (aRMR 1.33, 95%CI 0.99–1.79, *n* = 66) and Truck drivers (aRMR 1.37, 95% CI 0.84–2.22, *n* = 80) (Fig. 4).

The rates of COPD in the cohort were comparable to the general population rates and differed little by Work Category: Production (SMR 86, 95% CI 66–112; aRMR 0.94 95%CI 0.64–1.37, *n* = 54), Maintenance (SMR 99, 95% CI 74–135; aRMR 1.45, 95%CI 0.99–2.12, *n* = 42) and Construction (SMR 126, 95% CI 63–251; aRMR 1.51 95% CI 0.73–3.11, *n* = 8). Results for lower exposed workers are presented in Table 7.

**Table 7:** Relative mortality ratio (RMRs†) for male coal mine workers who only worked in administration, unexposed non-office and occasionally exposed jobs compared to the rest of the male cohort

Cause of Death Categories	ICD 10 Code	Unadjusted RMR (95% CI)	Adjusted* RMR (95% CI)
Only Administration			
All causes of death	A00 - Z99	0.97 (0.86–1.09)	**0.76 (0.68–0.86)**
All malignancies	C00–C97, D45–D46, D47.1, D47.3, D47.4, D47.5	1.22 (1.03–1.45)	0.84 (0.70–1.01)
All metabolic	E00-E99	1.37 (0.72–2.62)	0.97 (0.50–1.88)
All nervous system	G00 - G99	1.11 (0.48–2.55)	0.74 (0.32–1.69)
All circulatory	I00 – I99	0.92 (0.69–1.21)	0.65 (0.49–0.87)
All respiratory	J00 – J99	1.42 (0.88–2.31)	1.01 (0.62–1.64)
COPD	J40-J44	0.74 (0.32–1.67)	0.50 (0.22–1.13)
All digestive	K00-K93	1.26 (0.66–2.41)	1.05 (0.54–2.03)
All injury and trauma	V01-Y98	0.51 (0.38–0.69)	0.56 (0.42–0.76)
Accidents	V01-X59	0.40 (0.24–0.64)	0.43 (0.26–0.69)
Suicide	X60-X84	0.63 (0.42–0.94)	0.68 (0.46–1.03)
All other causes		1.06 (0.60–1.86)	0.80 (0.46–1.41)
Only Unexposed non-office			
All causes of death	A00 - Z99	1.31 (1.09–1.56)	1.15 (0.95–1.38)
All malignancies	C00–C97, D45–D46, D47.1, D47.3, D47.4, D47.5	1.25 (0.93–1.68)	1.07 (0.79–1.47)
All metabolic	E00-E99	1.30 (0.41–4.11)	1.14 (0.36–3.58)
All nervous system	G00 - G99	1.19 (0.29–4.85)	1.06 (0.26–4.36)
All circulatory	I00 – I99	1.83 (1.28–2.60)	**1.64 (1.15–2.35**)
All respiratory	J00 – J99	1.51 (0.67–3.40)	1.26 (0.57–2.81)
COPD	J40-J44	1.22 (0.39–3.82)	0.99 (0.32–2.99)
All digestive	K00-K93	2.04 (0.84–5.00)	1.89 (0.76–4.68)
All injury and trauma	V01-Y98	0.86 (0.56–1.30)	0.74 (0.48–1.14)
Accidents	V01-X59	0.94 (0.53–1.66)	0.81 (0.45–1.47)
Suicide	X60-X84	0.83 (0.45–1.55)	0.73 (0.39–1.37)
All other causes		1.59 (0.71–3.58)	1.16 (0.48–2.79)
Only Occasionally exposed jobs			
All causes of death	A00 - Z99	0.56 (0.47–0.66)	**0.80 (0.68–0.95)**
All malignancies	C00–C97, D45–D46, D47.1, D47.3, D47.4, D47.5	0.63 (0.49–0.81)	0.98 (0.75–1.27)
All metabolic	E00-E99	0.15 (0.02–1.10)	0.23 (0.03–1.68)
All nervous system	G00 - G99	0.88 (0.32–2.40)	1.27 (0.45–3.55)
All circulatory	I00 – I99	0.51 (0.34–0.76)	0.82 (0.55–1.22)
All respiratory	J00 – J99	0.18 (0.04–0.71)	0.33 (0.08–1.35)
COPD	J40-J44	0.14 (0.02–1.03)	0.28 (0.04–1.99)
All digestive	K00-K93	0.43 (0.14–1.36)	0.73 (0.23–2.32)
All injury and trauma	V01-Y98	0.58 (0.43–0.80)	0.64 (0.46–0.87)
Accidents	V01-X59	0.45 (0.28–0.74)	0.49 (0.29–0.80)
Suicide	X60-X84	0.74 (0.49–1.11)	0.81 (0.54–1.23)
All other causes		0.47 (0.20–1.16)	0.76 (0.31–1.87)

†Risk in each Work Category was compared to the rest of the male cohort

*Adjusted for era of first examination, age, smoking status, age & era interaction and age & smoking status interaction

Deaths from mesothelioma were increased among Maintenance Workers (SMR 168, 95%CI 101–278; aRMR 2.35, 95%CI 1.11–4.96, *n* = 15) and somewhat among Construction workers (SMR 228, 95%CI 73–706; aRMR 2.00 95%CI 0.61–6.59, *n* < 6), when compared with rates in the national population and the rest of the cohort. There were no other Work Categories with an excess of mesothelioma, there were fewer than six mesotheliomas among Production workers.

Increased mortality from all circulatory diseases was only observed among Labourers (Figs. 3 and 4). Ischaemic heart disease mortality was higher in Unexposed Non-office workers compared with the general Australian population (SMR 153, 95%CI 102–230, *n* = 23). After adjusting for age, era and smoking status, the within-cohort comparisons suggested that male Administration and Maintenance workers had a reduced risk of death from circulatory disease (Table 7; Fig. 4). However higher risks were seen for male Production workers, Unexposed Non-Office workers, Labourers and Truck Drivers (Table 7; Fig. 4).

Digestive disease mortality was raised for Construction workers compared to the rest of the cohort (aRMR 2.66, 95%CI 1.50–4.73, *n* = 14), mainly from liver disease (SMR 133, 95%CI 75–234, *n* = 12).

Stomach cancer mortality was higher in Occasionally Exposed workers (SMR 227, 95%CI 102–505, *n* = 6) while colorectal cancer mortality was higher in the Unexposed Non-Office group (SMR 229, 95%CI 123–426, *n* = 10) compared with the national population. Numbers of stomach and colorectal cancers were too small for meaningful internal comparisons.

Suicides were increased compared to the national population for men working in Production, Construction and as Truck Drivers (Fig. 3). However, this excess risk was attenuated (but still elevated when the cohort was compared with Queensland suicide rates) (Table 5). Internal comparisons within the cohort showed that the risk of suicide was similar across Work Categories (Fig. 4; Table 7).

An increased risk of mortality from accidents was seen in Cleaners, Truck drivers, Labourers, Exploration Drillers, Production and Construction workers when compared to Australian national population rates (Fig. 3; Table 5). However, comparison with Queensland-specific rates attenuated the increased risk for all these groups. The highest risks were for Cleaners, Truck drivers, Labourers, Exploration Drillers, Production and Construction workers (Table 5). Internal comparisons showed that male Administration, Occasionally Exposed and Maintenance workers were less likely to die from accidents than other coal mine workers, but the risk was higher for Production workers and Truck drivers (Table 7; Fig. 4).

## Discussion

In this study of mortality amongst a very large cohort of almost 190,000 coal mine workers, including over 160,000 men and nearly 24,000 women, there was reduced overall mortality in many but not all Work Categories. Mortality was reduced for Administration, Occasionally exposed, Maintenance and Production workers compared to the general population. This is common in industrial cohorts, likely attributable to the “healthy worker effect” [4, 12]. Jobs in coal mines, which require physically active workers, are less likely to recruit individuals with metabolic disorders such as diabetes or with respiratory diseases such as asthma and the SMRs for these disease groups were particularly low.

Deaths from circulatory disease were lower than that of the Australian population for all Work Categories except Labourers. It was significantly reduced for Maintenance, Supervisory and Production workers. When compared to the rest of the male cohort after adjusting for era, smoking and age, circulatory disease deaths were significantly increased among Production workers, Truck Drivers and Labourers (Unclear Work Category). This could be related to higher dust exposure in these jobs. Exposure to fine particulates in coal mine dust is associated with increased heart disease mortality [13, 14] as is exposure to diesel engine exhaust [15]. Long-term exposure to PM_2.5_ has been associated with overall mortality and with non-accidental, cardiovascular, and respiratory mortality even in Queensland, where ambient PM_2.5_ levels were typically well below the WHO air quality standard [16]. Studies of coal miners conducted in the USA have also shown higher cardiovascular/heart disease mortality, although none of the studies reported the jobs or categories of work of cohort members [17, 18]. Increased risks could be related to higher rates of high blood pressure and obesity among miners [19], something also reported in truck drivers in the USA [20]. Australian data show that Queensland truck drivers have poor eating and physical activity behaviours [21]. Individual data on these health and lifestyle factors were not available for the current cohort.

Lung cancer rates were higher for Production and Construction workers compared to the national population and the rest of the cohort even after adjusting for smoking. There was a higher risk for those with long exposure and those aged over 65 years, which suggests an occupational risk factor. The highest rate was for Drillers who are likely to have had the highest dust exposure.

When compared to the national population, the risk of death for non-malignant respiratory disease were not increased for most Work Categories, however the risks were higher for Maintenance and Construction workers compared to the rest of the cohort after adjusting for age, era and smoking. There were only six deaths attributed to dust diseases such as pneumoconiosis, insufficient numbers to analyse by Work Category. Internal mortality analyses showed that among Administration workers, the overall mortality risk decreased after adjustment for age, smoking and era, suggesting some of the observed excess mortality could be a result of differential smoking rates.

Deaths resulting from mesothelioma were higher in the Maintenance and likely among Construction workers when compared with the national population and also with the rest of the cohort. Historically, both of these groups of workers were likely to be occupationally exposed to asbestos. It has been found that the most common jobs carried out by those diagnosed with mesothelioma in Australia between 2010 and 2018 were maintenance work including in construction, electricians and fitters [22]. 

Digestive disease mortality, mainly from liver disease was raised for Construction workers compared to the general population. We had no information on alcohol consumption and so could not adjust for this.

Suicide rates in some of the Work Categories were higher than the general Australian population rates, but attenuated when compared to Queensland State rates. Increased deaths by suicide have been reported internationally in the mining sector for many years, including reports in the 1930 s and 1960 s [23] and in a more recent study from the USA [24] and from Australia [25]. Comparing adjusted suicide rates across Work Categories showed that risks were similar, suggesting that there were common risks for most groups of mine workers. A number of reasons for higher suicide rates in miners have been postulated, mainly related to factors such as work with low job control and high demands, perceived job insecurity, shift-work or atypical hours and isolation resulting from long distance commuting, and workers being reluctant to seek help when in distress as a result of a culture of traditional masculine norms and/or there could be a culture of alcohol and drug use [26]. 

Cleaners and Truck Drivers were at somewhat higher risk of accidental death than the general population, even when compared with Queensland population rates. The death data did not indicate whether the accidents were work-related, but it was unlikely that many of the cohort’s accidental deaths were from workplace accidents. SafeWork Australia (the Australian national regulatory body) reports identified 20 fatalities in mining in Queensland between 2013 and 2021 [27, 28]. Not all of these deaths would have been in coal mining. Queensland is a large state with an area of over 1,700,000 km^2^. Long travel distances to and from worksites or for leisure activities would increase the risks of motor vehicle accidents.

It is interesting that Supervisors in the Unclear Work Category had the lowest SMRs and RMRs for all major causes of death. This could be a survivor effect as they are likely to be experienced in order to become supervisors.

The strengths of our study include the near complete list of the large number of Queensland coal mine workers employed since 1993, including 24,389 women. Smoking data, available from at least one assessment for over 99% of participants, were a major asset. However, measurement error cannot be ruled out, for example, a good intention on the morning of the examination might record individuals as ex-smokers, but they subsequently continue to smoke. The presence of several assessments for some workers should limit this problem. Higher lung cancer mortality was observed for Production workers and this did not attenuate when adjusted for smoking in internal analyses, perhaps suggesting that this is not smoking related.

Workers who only had assessments before 1993 did not have recorded job titles, so they could not be included in the analyses by Work Category. The cohort was only followed-up commencing from 1993, rather than date of first employment to avoid any introduction of an immortal time bias. However, only about 2% of the cohort had no recorded job title, so the majority of the cohort were included in these analyses.

The Work Categories were checked with industry representatives and experts. However, there may be some residual misclassification resulting from categorising the free text. Where possible, Industrial cleaners were grouped with maintenance workers, office cleaners with unexposed workers, Truck drivers, Supervisors and Labourers were grouped with Maintenance or Production. Those who could not be allocated were grouped into the “Unclear” Work Category. The job title was also only captured at each 5 year assessment, so job movement in between these times was not recorded. However, we believe that this study is the first to look in depth at job-specific rates of mortality among mine workers.

Matching the cohort names and birth dates to those in the NDI was a probabilistic process. The availability of middle names and dates of last known employment from RSHQ records improved the probability of a correct match to the NDI. Previous validation studies of the NDI have found good sensitivity and specificity of the matching process [29, 30]. 

The limitations of the study include the relative youth of the cohort and the short follow-up period, starting in 2000 for the majority of workers commencing work, particularly for women, as 60% of women had their first assessment in or after 2010. This means that fatal diseases with a long latent period may not (yet) have caused death. The study was sufficiently powered to identify significantly increased risks of most major death categories for the male but not for the female workers.

The number of calculated risk estimates was large in this study. For example, for external analyses, there were ten major categories of cause of death, and an overall risk estimate, for both men and women. These multiple analyses mean that it is likely that one or more of these may be a chance finding [31]. Some of the Work Categories, especially for women are small so that one or two deaths included or excluded by chance could have affected the point estimate.

Changes in job title could have occurred, where these were documented in assessments, we categorised people as ONLY Administration, ONLY Unexposed Non-Office or ONLY Occasionally exposed. All other categories were ever held a job in that work Category.

The exact dates of employment as a coal mine worker were unavailable as were data on other possible confounding factors such as ethnicity, alcohol consumption, physical activity, genetic predisposition and non-occupational exposures. However, the availability of smoking data allowed for this major confounder to be accounted for in within-cohort analyses and this study covers one of the largest coal mine worker cohorts to be investigated contemporaneously.

The majority of this cohort were young, but mortality varied between Work Categories, with excess mortality observed particularly in Production, Labourers, Truck drivers and Construction among male workers and among women Cleaners. Increased risks of mortality were seen for circulatory diseases, accidents and respiratory diseases. Risk of suicide has varied over the years and the increase is attenuated when compared to Queensland rates. The “healthy worker effect” likely played a crucial role, with reduced mortality for many causes of death in some of the Work Categories.

Given the age of most workers and the short observation period from 2010 the end of 2020 for many workers recently commencing work, another follow-up in 5 to 10 years will provide more insight into possible work-related mortality.

## Supplementary Information

Supplementary Material 1.

## Data Availability

Health data has been obtained from a National repository and is not available to other researchers. Coal mine worker identifiers were obtained from Queensland government Department RSHQ. Original data are not available as a result of Australian privacy legislation and terms of our Ethics approvals.

## Abbreviations

95% CI: 95% Confidence interval
aRMR: Relative mortality ratios (adjusted for era, age and smoking status)
ABS: Australian Bureau of Statistics
AIHW: Australian Institute of Health and WEelfare
CHPP: Coal Handling and Preparation Plant
CI: Confidence interval
COPD: Chronic obstructive lung disease
E: Expected
ERZ: Explosion Risk Zone controller
ICD-10: International Classification of Disease version 10
nec: Not elsewhere classified
NHMRC: National Health and Medical Research Council
O: Observed
OCE: Open Ccut Examiner
PY: Person-years
RMR: Relative mortality ratio
RSHQ: Resources, Safety and Health Queensland
SMR: Standardised mortality ratio compared to Australian national rates
SMR_Q_: Standardised mortality ratio, compared to Queensland rates
