# Supplementary material for: Are mortality rates similar between jobs in the Queensland coal mine workers’ cohort?
Source: BMC Public Health. 2025 Sep 1;25:3004. doi: 10.1186/s12889-025-24163-4 (PMC12400750; doi:10.1186/s12889-025-24163-4)
Supplement: Supplementary file 1 — Supplementary Material 1. [file 12889_2025_24163_MOESM1_ESM.docx]

Supplementary File

Description of Job Groups included within each Work Category

| **Work Category** | | **Job Groups** | **Number who only/ever (%) worked in the Job Group or Work Category** |
| --- | --- | --- | --- |
| ONLY Unexposed Office | | Administration | 20134/31818 (63.3%) |
| ONLY Unexposed Non-Office | | Blue Collar (Not Exposed) | 3932/6184 (63.6%) |
|  |  | Control Room | 213/765 (27.8%) |
|  |  | Environmental Services | 2398/3514 (68.2%) |
|  |  | All unexposed Non-Office workers | 6543/10419 (62.8%) |
| ONLY Occasionally exposed | | Engineer | 56529/10108 (64.6%) |
|  |  | Mine Services | 1609/2910 (55.3%) |
|  |  | Technical Services (Including Geologist, Surveyor) | 4450/6334 (70.3%) |
|  |  | All occasionally exposed workers | 12588/18959 (66.4%) |
| EVER Maintenance- all | | Maintenance – General | 8558/20027 (42.7%) |
|  |  | Abrasive, Blast, Sand, Paint | 407 (53.1%) |
|  |  | Shutdown Maintenance | 4109/6236 (65.9%) |
|  |  | Belt Splicer | 270/573 (47.1%) |
|  |  | Boilermaker | 5817/8298 (70.1%) |
|  |  | Fitter (Nec) * | 5431/12765 (42.6%) |
|  |  | Diesel Fitter | 4506/10222 (44.1%) |
|  |  | Tyre Fitter | 377/689 (54.7%) |
|  |  | Electrician | 8196/11411 (71.8%) |
|  |  | Electrician (Auto) | 756/1192 (63.4%) |
|  |  | Industrial Cleaner | 836/1547 (54.0%) |
|  |  | All Maintenance workers | 458202/63334 (71.4%) |
| EVER Production- all | Production – General | 20/78 (25.6%) |  |
|  | Blast Crew | 708/2025 (35.0%) |  |
|  | Driller (Blast) | 74/397 (18.6%) |  |
|  | Driller (General) | 1911/3877 (49.3%) |  |
|  | CHPP Plant Operator | 224/1597 (14.0%) |  |
|  | Laboratory | 945/1649 (57.3%) |  |
|  | Driller (Ug, Coal Seam) | 104/268 (38.8%) |  |
|  | Explosion risk zone (ERZ) controller/ Deputy | 171/1039 (16.5%) |  |
|  | Open cut Examiner | 50/548 (9.1%) |  |
|  | Dragline | 51/1054 (4.8%) |  |
|  | Secondary Support | 126/333 (37.8%) |  |
|  | Miner | 2874/10813 (26.6%) |  |
|  | Operator (Inc Mobile Operator, Production Operator, Production Truck Driver) | 26034/48246 (54.0%) |  |
|  | Prestrip | 49/501 (9.8%) |  |
|  | Scraper | 65/176 (36.9%) |  |
|  | Production Support | 7/25 (28.0%) |  |
|  | Tunneller | 21/58 (36.2%) |  |
|  | All Production workers | 38147/62165 (61.4%) |  |
| EVER Exploration | Driller (Exploration) | 3236/5013 (64.6%) |  |
| EVER Construction | Civil Works | 329/806 (40.8%) |  |
|  | Construction | 6093/8355 (72.9%) |  |
|  | All Construction workers | 6426/9147 (70.3%) |  |
| Work Category Unclear | EVER Labourer (nec) * | 3245/6059 (53.6%) |  |
|  | EVER Cleaner (nec) * | 2483/3711 (66.9%) |  |
|  | EVER Supervisor (nec) * | 1495/5641 (26.5%) |  |
|  | EVER Truck Driver (nec) * | 4314/8595 (50.2%) |  |

* NEC is not elsewhere classified
